# Elevated Vascular Sympathetic Neurotransmission and Remodelling Is a Common Feature in a Rat Model of Foetal Programming of Hypertension and SHR

**DOI:** 10.3390/biomedicines10081902

**Published:** 2022-08-05

**Authors:** Maria Sofia Vieira-Rocha, Joana Beatriz Sousa, Pilar Rodríguez-Rodríguez, Silvia Madaglena Arribas, Carmen Diniz

**Affiliations:** 1 Laboratory of Pharmacology, Department of Drug Science, Faculty of Pharmacy, University of Porto, 4050-313 Porto, Portugal; 2 LAQV/REQUIMTE, Faculty of Pharmacy, University of Porto, 4050-313 Porto, Portugal; 3 Department of Physiology, Faculty of Medicine, Universidad Autonoma de Madrid, 28029 Madrid, Spain

**Keywords:** foetal programming of hypertension, sympathetic neurotransmission, sympathetic innervation, vascular remodelling, fibrosis, foetal undernutrition

## Abstract

Hypertension is of unknown aetiology, with sympathetic nervous system hyperactivation being one of the possible contributors. Hypertension may have a developmental origin, owing to the exposure to adverse factors during the intrauterine period. Our hypothesis is that sympathetic hyperinnervation may be implicated in hypertension of developmental origins, being this is a common feature with essential hypertension. Two-animal models were used: spontaneously hypertensive rats (SHR-model of essential hypertension) and offspring from dams exposed to undernutrition (MUN-model of developmental hypertension), with their respective controls. In adult males, we assessed systolic blood pressure (SBP), diastolic blood pressure (DBP), heart rate (HR), sympathetic nerve function (^3^H-tritium release), sympathetic innervation (immunohistochemistry) and vascular remodelling (histology). MUN showed higher SBP/DBP, but not HR, while SHR exhibited higher SBP/DBP/HR. Regarding the mesenteric arteries, MUN and SHR showed reduced lumen, increased media and adventitial thickness and increased wall/lumen and connective tissue compared to respective controls. Regarding sympathetic nerve activation, MUN and SHR showed higher tritium release compared to controls. Total tritium tissue/tyrosine hydroxylase detection was higher in SHR and MUN adventitia arteries compared to respective controls. In conclusion, sympathetic hyperinnervation may be one of the contributors to vascular remodelling and hypertension in rats exposed to undernutrition during intrauterine life, which is a common feature with spontaneous hypertension.

## 1. Introduction

Hypertension is one of the most important risk factors of cardiovascular disease, and despite the current treatment options, a substantial portion of the population still have uncontrolled or suboptimal controlled blood pressure (BP) [1]. Additionally, the aetiology of hypertension remains unknown in most cases. Sympathetic nervous system (SNS) has an integral role in the regulation of heart rate and contractility, vascular tone and fluid volume. SNS hyperactivation leads to retention of salt and/or water and increases cardiac output and peripheral resistance [2]. In addition, elevated SNS also participates in vasculokar remodelling related to smooth-muscle hypertrophy and fibrosis [3], contributing to the development and/or the maintenance of hypertension through an increase in peripheral resistance [4,5,6]. Recent advances have led to the understanding that hypertension may have a developmental origin. It is now well accepted that the foetus can adapt to adverse intrauterine conditions promoting physiological alterations in foetal development to ensure survival [7]. Such alterations, later in life, may increase the susceptibility to develop hypertension and cardiometabolic diseases, in a process named as foetal programming [8,9,10]. Several adverse factors during intrauterine life have been demonstrated to contribute to inadequate foetal development and foetal programming. The most important ones are malnutrition [9,11], oxygen deprivation [12,13,14], placental insufficiency [15], exposure to excess of glucocorticoids [16,17,18], toxic substances (alcohol, tobacco) [19,20,21] and environmental pollutants [22]. The mechanisms underlying an offspring’s predisposition to develop hypertension in adulthood have not been completely addressed. Nevertheless, implication of increased oxidative stress [23,24], alterations in the glucocorticoid axis [16,25,26,27] or activation of the renin-angiotensin system (RAS) [28,29,30] have been suggested. Some of these alterations might be mediated by epigenetic modulation of genes implicated in cardiovascular control [24,31,32,33,34] and/or alterations in renal or vascular autonomic functions [14,26,35,36,37,38]. In the context of foetal programming hypertension (FPH), the contribution of the peripheral sympathetic nervous system is still not completely understood [39]. The spontaneously hypertensive rats (SHR), developed by Okamoto and Aoki 1963 [40] is a well-established model, which resembles essential hypertension in humans [41]. Among other common features, the SHR exhibits alterations in RAS [42], sympathetic hyperinnervation [43] and resistance artery remodelling [44], features also present in human essential hypertension. SHR and rat models of FPH also share some similarities regarding blood pressure development, i.e., the sexual dimorphism and the time course of development [45,46].

In the current work, we hypothesize that hypertension of developmental origin may share features with SHR regarding sympathetic hyperinnervation, which may contribute to vascular remodelling and hypertension development. Our aims were to evaluate, in mesenteric arteries from SHR and from a rat model of FPH induced by maternal undernutrition during gestation (MUN), the (i) sympathetic innervation, (ii) sympathetic activation and (iii) vascular remodelling.

## 2. Materials and Methods

### 2.1. Animals

Sprague–Dawley, Wistar Kyoto (WKY) and spontaneously hypertensive (SHR) rats from the colony maintained at the animal house facility of the Universidad Autónoma de Madrid were used. All experimental procedures were approved by the Ethics Review Board of Universidad Autónoma de Madrid and Comunidad Autónoma de Madrid (CEI63-1112-A097 and PROEX 04/19) according to the Guidelines for the Care and Use of Laboratory Animals (National Institutes of Health publication no. 85-23, revised in 1996), the Spanish legislation (RD 1201/2005) and the Directive 2010/63/EU on the protection of animals used for scientific purposes. The rats were housed in buckets 36.5/21.5/18.5 cm (length/width/height) on aspen wood bedding, under controlled conditions of 22 °C, 40% relative humidity and 12/12 light/dark photoperiod. The animal health monitoring indicated that they were free from pathogens that may interact with any of the parameters studied. The ARRIVE Guidelines were followed for reporting in vivo experiments [47].

#### 2.1.1. Experimental Model of FPH

FPH model based on global maternal nutrient restriction was induced as previously described [46,48]. Two study groups were established using the Sprague–Dawley strain: a CONTROL group, with ad libitum feeding throughout pregnancy and lactation, and a group with intake restriction during part of gestation (maternal undernutrition model, MUN). This last group of rats had ad libitum diet during the first half of the gestation (from day 1 to 10), and then they were fed with 50% of the intake of a pregnant rat (from day 11 until delivery). The maximum daily intake of rat chow was previously determined in a group of pregnant rats as 24 g/day. After delivery and through the lactation period, the mothers were fed ad libitum. The mothers were fed with a breeding diet (Euro Rodent Diet 22; 5LF5, Labdiet, Madrid, Spain) containing 55% carbohydrates, 22% protein, 4.4% fat, 4.1% fibre and 5.4% mineral at 12.2% humidity. Drinking water was provided ad libitum to all animals. Immediately after birth, the offspring were weighed individually and sexed, and the litter was randomly standardised to 12 rats, 6 males and 6 females, if possible. The rest of the litter was sacrificed with CO_2_. At the age of 6 months, male offspring from the two experimental groups (control and MUN) were analysed.

#### 2.1.2. Experimental Model of Spontaneous Hypertension

SHR, a well-known animal model of essential hypertension, was also used to make comparisons with the animal model of FPH induced by foetal undernutrition. Wistar-Kyoto rats (WKY) were chosen as the control model, representing a normotensive state. Both WKY and SHR male rats were bred at the Animal House of Universidad Autónoma de Madrid and used at the age of 6 months.

#### 2.1.3. Experimental Protocol

The animals from the different experimental groups (WKY, SHR, CONTROL and MUN) were first weighed and then anesthetized to measure the haemodynamic parameters (see below). Thereafter, the rats were sacrificed using a guillotine, the method being reported as the sacrificial advisable in studies involving the nervous system. The tissue samples were collected immediately after sacrifice and the mesenteric bed collected from which the main mesenteric artery was dissected. Four segments were obtained from each tissue.

### 2.2. Chemicals

The following drugs were used: levo-[ring-2,5,6-3H]-noradrenaline, specific activity 44.8 Ci/mmol (DuPont NEN, I.L.C., Lisboa, Portugal); the scintillation mixture used was from OptiPhase ‘Hisafe’ 3, PerkinElmer, I.L.C. (Lisboa, Portugal); desipramine hydrochloride purchased from Sigma-Aldrich (Sintra, Portugal). Entellan (mounting medium), Orcein, Masson’s trichrome and haematoxylin/eosin from Merck (Darmstadt, Germany). The following antibodies were used: mouse monoclonal anti-tyrosine hydroxylase antibody (ab137869), from Abcam, London, UK) and Alexa Fluor 488 goat anti-mouse IgG (H + L) antibody, highly cross-adsorbed (Invitrogen, Life Technologies, SA, Madrid, Spain); vectashield mounting medium with DAPI (Vector Laboratories, London, UK). Stock solutions were made up in ultrapure water and diluted in superfusion medium immediately before use.

### 2.3. Haemodynamic Parameters Measurement

Haemodynamic parameters were determined as previously described [49]. Briefly, the rats were anesthetized (37.5 mg/kg Ketamine hydrochloride and 0.25 mg/kg Medetomidine hydrochloride i.p.). Thereafter, a cannula was inserted into the right iliac artery and connected to a Pressure transducer (Statham, Harvard Apparatus GmbH, Berlin, Germany). The blood pressure wave was recorded on a PC computer, using the data acquisition with the PowerLab system (ADInstruments) for 60 min and systolic blood pressure (SBP), diastolic blood pressure (DBP) and heart rate (HR) were measured in the final portion of the recorded trace.

### 2.4. [^3^H]-Noradrenaline Release Experiments

Evaluation of [^3^H]-noradrenaline (NA) release experiments was carried out as previously described [50,51,52]. Arteries were preincubated in 2 mL of Krebs-Henseleit solution containing 0.1 µmol/L [^3^H]-NA (for 60 min at 37 °C) and transferred into superfusion chambers, superfused with [^3^H]-NA-free medium (1 mL/min; constant rate: Krebs-Henseleit solution with desipramine 400 nmol/L to inhibit NA’s neuronal uptake). Three identical periods of electrical stimulation were applied (Hugo Sachs Elektronik, March-Hugstetten, Germany; constant current mode, rectangular pulses; 1 ms, with current strength 50 mA; 5 Hz, 100 pulses). The first, starting at t = 30 min (S0) was not used for determination of tritium outflow. The subsequent periods (S_1_ and S_2_) were applied at t = 90 min and t = 120 min, respectively. The superfusate was collected each 5-min period, starting from minute 85 of superfusion onwards. At the end of the experiments (t = 130 min), tritium was measured in superfusate samples and solubilized arteries (sonicated 1 h with 2.5 mL of 0.2 mol/L perchloric acid) by liquid scintillation spectrometry (LS 6500, Beckman Instruments, Fullerton, CA, USA) after adding 6 mL of a scintillation mixture to each sample.

Electrically evoked tritium overflow from artery segments incubated with [^3^H]-NA was shown to reflect action potential-evoked neuronal NA release and evoked tritium overflow are assumed to reflect changes in neuronal NA release [43,50,53]. Values of [^3^H]-NA uptake were estimated as previously described [54]: the tissue tritium content was obtained at the end of each [^3^H]-NA release experiment and was summed to values of [^3^H]-NA previously collected in the 5-min superfusate samples (from t = 85 min to t = 130 min, control segments). The final value was considered as the total amount of incorporated [^3^H]-NA in individual mesenteric artery segments (total tissue tritium content).

### 2.5. Immunohistochemistry

Immunohistochemistry procedures were previously described [53]. Briefly, four artery segments were obtained from each artery and immediately placed in cold phosphate buffer solution (PBS; in g/L): NaCl 8.0, Na_2_HPO_4_.2H_2_O 0.77, KCl 0.20 and KH_2_PO_4_ 0.19 (pH 7.2). Each segment was longitudinally opened and fixed (paraformaldehyde 4% PBS; 50 min; room temperature). After two 15-min PBS washing cycles, artery segments were incubated with the primary antibody (mouse monoclonal anti-tyrosine hydroxylase, TH, 1:100 dilution, overnight, 4 °C to stain noradrenergic nerve terminals). Thereafter, tissues were incubated with Alexa 488 anti-mouse fluorescent secondary antibody (1:1000 dilution, 1 h, room temperature). The primary antibody was previously validated by the manufacturer. Negative controls were incubated on adjacent sections using 10% normal horse serum or blocking solution instead of the primary antibody. After three PBS washing cycles, the arteries were mounted intact with antifading agent (headshield mounting medium with DAPI).

Artery segments were visualized with a Leica SP2 laser scanning confocal microscopy (LSCM) system (Leica Microsystems, Metzler, Germany) fitted with an inverted microscope (×63 oil immersion lens). Stacks of 1-μm-thick serial optical images were captured from five randomly chosen regions along the adventitial layer of the mesenteric artery, which was identified by the shape and orientation of the nuclei stained with DAPI [55]. Adventitia was scanned along each mesenteric artery and the resulting images were reconstructed separately for each wavelength. Two stacks of images were sequentially obtained at different wavelengths: the first stack was taken with the Ex 405 nm and Me 412–470 nm wavelength to visualize cell nuclei (DAPI staining). The second was taken with the Ex 488 nm and Em 490–570 nm wavelength to visualize the TH (location of sympathetic terminals). Image acquisition was always performed under the same laser power, brightness, and contrast conditions. The resulting images were reconstructed separately for each wavelength for later quantification.

Quantitative analysis of confocal z-stacks images was performed using image analysis software (PAQI, CEMUP, Porto, Portugal) as previously described [56]. Briefly, a sequential routine was designed and developed to analyse each fluorescent signal used. PAQI software measured the surface area and strength of the fluorescence signal marking the postganglionic sympathetic nerves.

### 2.6. Histology

Serial 2-µm thickness sections of mesenteric arteries, previously fixed in paraformaldehyde 4% PBS, were dewaxed in xylene, then they were hydrated in decreasing concentrations of alcohols and stained with orcein, haematoxylin/eosin and with Masson Trichrome. Each tissue was cut in five levels along the length of the vessel to ensure data represents the putative mesenteric artery heterogeneity rather than only a specific location of the artery. Each batch represents histochemical staining and includes sections from all five levels of mesenteric artery from each animal group. This procedure was repeated three times (3 batches). In total, 150 sections were obtained. Sections were stained and divided according to animal source. Within each of these groups, a random selection of the sections was carried out.

Stained sections were visualized using a high-resolution Zeiss Axiocam 105 colour digital camera mounted on a Zeiss Primo Star microscope, using an ×10 objective, to analyse the arterial lumen, media and adventitia layer. Histomorphometry was performed with ImageJ software [57], and data of lumen diameter and cross sectional area of the media and the adventitia were obtained.

### 2.7. Statistics

Statistics were performed with GraphPad Prism (version 8.3) software (San Diego, CA, USA). Sample size was calculated assuming a probability error of alpha type of 5% (*p* < 0.05) and potency of 80%. The normality of the variables was evaluated with Kolmogorov-Smirnov test. Results were expressed as mean ± s.e.m. Differences of means were compared using one- or two-way ANOVA, followed by post-hoc Holm-Sidak’s multicomparison *t* test or Student’s *t* test. A *p* value lower than 0.05 was considered to denote statistically significant differences.

## 3. Results

Body weight was not different between male MUN (460.18 ± 8.1 g; *n* = 6) and CONTROL rats (486.6 ± 18.6 g; *n* = 6; *p* = 0.21). Moreover, no differences between body weight of SHR (377.4 ± 8.9; *n* = 6) and WKY (367.0 ± 11.9 g; *n* = 6, *p* = 0.52) were found.

MUN exhibited larger SPB and DBP, but not HR, when compared to CONTROL, whereas SHR evidenced larger SBP, DBP and HR when compared to WKY rats (Table 1). MUN showed lower SBP and DBP levels compared to SHR. However, HR was not enhanced in MUN, contrasting with the HR values observed in SHR.

### 3.1. Influence of Foetal Undernutrition on Vascular Morphology

**Figure 1:**
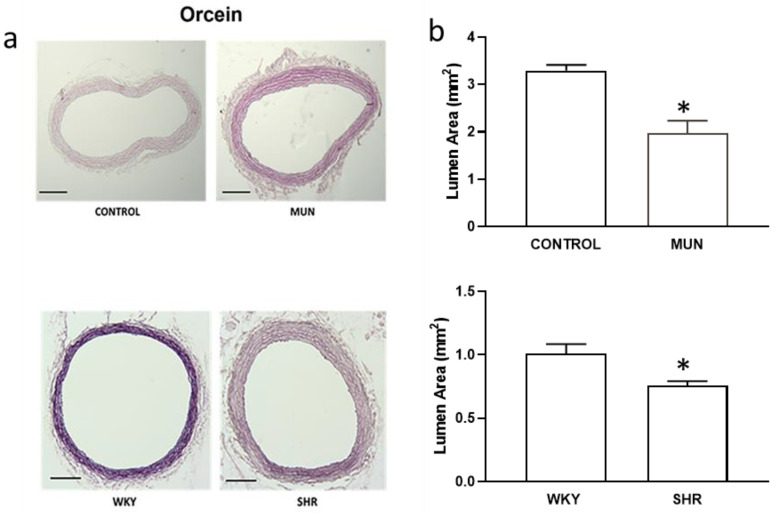
Histomorphometry of the lumen of mesenteric arteries from CONTROL and MUN (upper panel) and WKY and SHR rats (lower panel). (**a**) Images were obtained from orcein stained arteries (scale bar = 500 µm); (**b**) the graphics show the lumen area. MUN, offspring exposed to maternal undernutrition during pregnancy; CONTROL, offspring from mothers fed ad libitum during pregnancy; SHR, spontaneously hypertensive rats; WKY, Wistar Kyoto rats. Values are mean ± s.e.m. from 6 rats of each group. Significant differences from the respective control rat: * *p* < 0.05.

**Figure 2:**
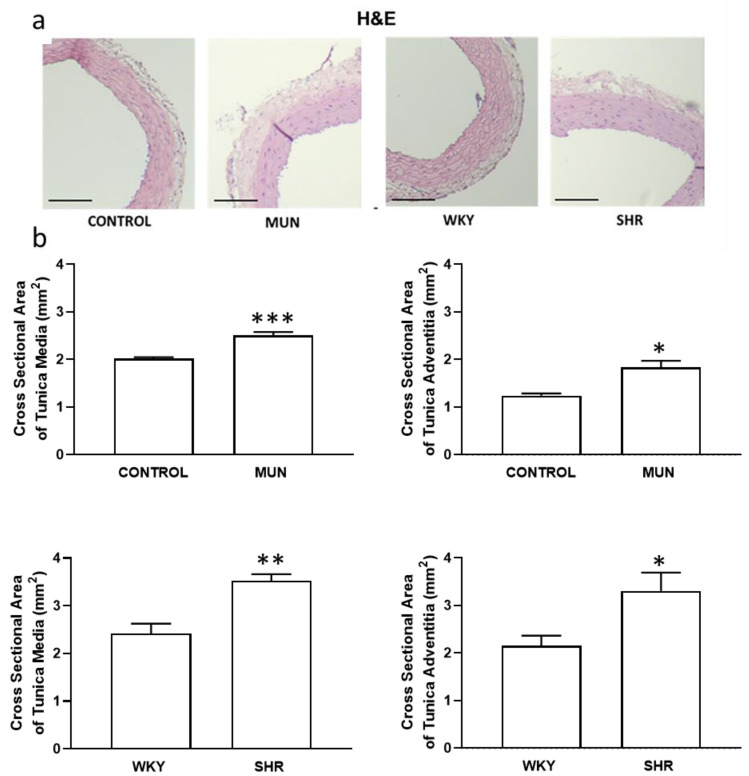
Histomorphometry of hypertensive (MUN and SHR) and normotensive (CONTROL and WKY) mesenteric wall. (**a**) Images were obtained from haematoxylin/eosin-stained arteries (scare bar = 300 µm). (**b**) Cross-sectional area of tunica media (right panel) and cross-sectional area of tunica adventitia (left panel). MUN, offspring exposed to maternal undernutrition during pregnancy; CONTROL, offspring from mothers fed ad libitum during pregnancy; SHR, spontaneously hypertensive rats; WKY, Wistar Kyoto rats. Values are mean ± s.e.m. from 6 rats of each group. Significant differences from the respective control rat: * *p* < 0.05; ** *p* < 0.01 *** *p* < 0.001.

Histological sections of the mesenteric artery showed a reduced lumen area in MUN compared to CONTROL (Figure 1a and Figure 2b), a reduction of MUN arterial lumen near 0.6-fold relatively to CONTROL values. Such effect was also observed in arteries from SHR and WKY, a reduction of SHR arterial lumen near 0.7-fold relatively to WKY values.

The media and adventitia layers of MUN were thicker compared to CONTROL (with an increase near 1.3-fold and 1.5-fold, respectively) similarly to what occurred in SHR compared to WKY (with an increase near 1.4-fold and 1.5-fold, respectively) (Figure 2a,b). Moreover, ratios of media/lumen and of adventitia/lumen were significantly increased in both MUN and SHR compared to data from respective controls, CONTROL and WKY (Table 2).

**Figure 3:**
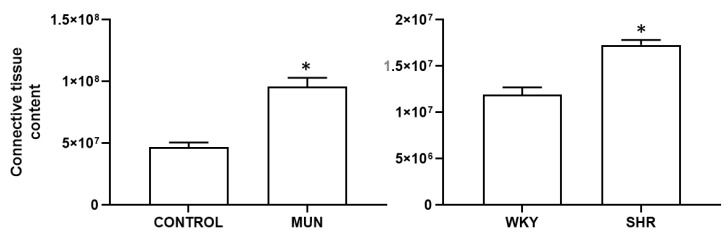
Histomorphometry of hypertensive (MUN and SHR) and normotensive (CONTROL and WKY) mesenteric arteries, stained with Trichrome. MUN, offspring exposed to maternal undernutrition during pregnancy; CONTROL, offspring from mothers fed ad libitum during pregnancy; SHR, spontaneously hypertensive rats; WKY, Wistar Kyoto rats. Values are mean ± s.e.m. from 6 rats of each group. Significant differences from the respective control rat: * *p* < 0.05.

An increase in the connective tissue content was also observed, both in MUN and SHR, compared to their respective controls (CONTROL and WKY, respectively, Figure 3); the connective tissue increased in MUN near 2.1-fold relatively to CONTROL, whereas in SHR, it increased 1.5-fold relatively to WKY).

### 3.2. Influence of Foetal Undernutrition in Sympathetic Postganglionic Nerves Activation

**Figure 4:**
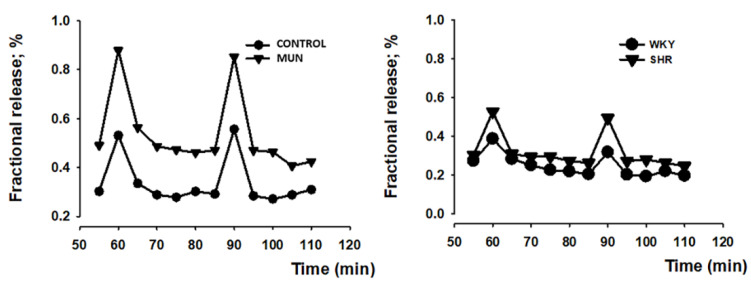
Representative examples of time course tritium outflow from: mesenteric arteries from normotensive animals, CONTROL (circles, left panel) and WKY (circles, right panel), and hypertensive animals, MUN (triangles, left panel) and SHR (triangles, right panel) from typical experiments. After pre-incubation with [^3^H]-noradrenaline, tissues were superfused with [^3^H]-noradrenaline-free medium containing desipramine (400 nM). Tritium outflow (ordinates) is expressed as a percentage of the total radioactivity present in the tissue at the beginning of the collection period and was measured in samples collected every 5 min. Artery segments were stimulated twice by 100 pulses/5 Hz, (S_1_, S_2_). Each line represents the outflow of tritium from a single superfusion chamber; MUN, offspring exposed to maternal undernutrition during pregnancy; CONTROL, offspring from mothers fed ad libitum during pregnancy; SHR, spontaneously hypertensive rats; WKY, Wistar Kyoto rats.

Electrical field stimulation (5 Hz, 1 ms, 100 pulses, 50 mA) tritium outflow was higher in mesenteric arteries from MUN compared to CONTROL (Figure 4). Similarly, there was a larger tritium outflow in mesenteric arteries from SHR compared to WKY (Figure 4). The fractional rate of basal tritium outflow (b_1_), electrically evoked tritium overflow (S_1_) and S_2_/S_1_ ratios are shown in Table 3. Basal outflow and electrically evoked tritium overflow remained constant throughout the control experiments, with b_n_/b_1_ and S_n_/S_1_ values close to unity. Electrically evoked tritium overflow (S_1_) was higher in hypertensive arteries (both SHR and MUN) compared to their respective control (WKY or CONTROL) vessels (Table 3). However, the evoked overflow was similar between the hypertensive arteries (MUN *versus* SHR arteries: *p* > 0.05).

### 3.3. Influence of Foetal Undernutrition on Perivascular Sympathetic Innervation

**Figure 5:**
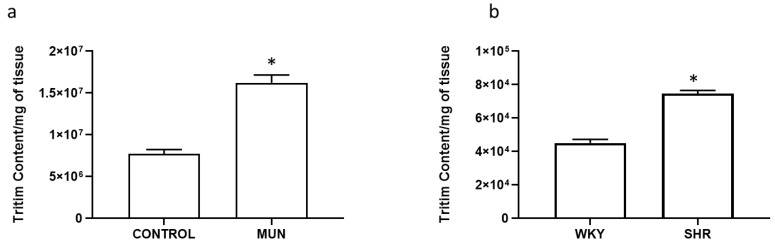
[^3^H]-Tritium uptake in mesenteric arteries from (**a**) CONTROL and MUN and (**b**) WKY and SHR rats. MUN, offspring exposed to maternal undernutrition during pregnancy; CONTROL, offspring from mothers fed ad libitum during pregnancy; SHR, spontaneously hypertensive rats; WKY, Wistar Kyoto rats. Values are mean ± s.e.m. from 6 animals per group. Significant differences from the respective control rats: * *p* < 0.05.

In our experimental conditions, total tissue tritium content (per mg of tissue) was higher in MUN compared to CONTROL arteries (Figure 5a): total tissue content in MUN increased near 2.1-fold relatively to CONTROL. Moreover, tritium uptake was also larger in SHR mesenteric arteries compared to the values from WKY arteries (Figure 5b): total tissue content in SHR increased 1.7-fold relatively to WKY.

**Figure 6:**
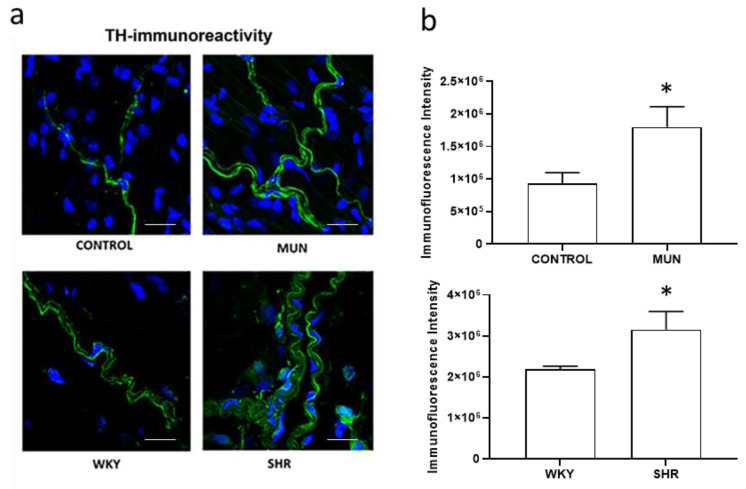
(**a**) Laser scanning confocal microscopy representative images of the adventitia layer of mesenteric arteries from CONTROL and MUN (upper panel) WKY and SHR (lower panel) rats. Images show the immunofluorescence reactivity to TH (green) with DAPI-stained nuclei (blue). Scale bar: 25 μm. (**b**) Quantitative analysis of LSCM images. MUN, offspring exposed to maternal undernutrition during pregnancy; CONTROL, offspring from mothers fed ad libitum during pregnancy; SHR, spontaneously hypertensive rats; WKY, Wistar Kyoto rats. In the graphics, values are mean ± s.e.m. from 6 rats from each group. Significant differences from the respective control rats: * *p* < 0.05.

The influence of foetal undernutrition on perivascular sympathetic innervation in the adventitial layer of mesenteric arteries (identified from LSCM images by the shape and orientation of the nuclei and by exhibiting scattered fibres [56], was also assessed using a sympathetic neuronal marker, thyroxine hydroxylase (TH). Non-significant immunoreactivity was observed when the primary antibody was omitted (negative controls, data not shown). In mesenteric arteries from all experimental groups, immunoreactivity for the sympathetic neuronal marker TH evidenced the presence of sympathetic nervous fibres (Figure 6a, green marker). However, the pattern of TH immunoreactivity in arteries from SHR and from MUN exceeded those observed in WKY and CONTROL arteries, respectively (Figure 6b), revealing a denser and thicker sympathetic innervation: TH immunoreactivity, in MUN, increased near 1.9-fold relatively to CONTROL, whereas, in SHR, it increased 1.5-fold relatively to WKY.

## 4. Discussion

The current study shows that foetal undernutrition induces an increase in the arterial sympathetic neurotransmission in rat adulthood (6-months old) which can justify, at least in part, the arterial inward hypertrophic remodelling observed and further highlighted the role of adventitia in the pathophysiology of foetal programming of hypertension. In addition, this study also shows that MUN rats share with SHR, a rat model of essential hypertension, these pathological aspects, which may contribute to the development of hypertension.

Foetal undernutrition was associated with the development of sympathetic hyperinnervation observed in the adventitial layer of the mesenteric artery. LSCM data revealed the presence of nerve fibres positive for TH (a sympathetic nervous fibre marker), spread through the adventitia reaching the medial layer, being thicker in MUN mesenteric arteries. Tyrosine hydroxylase (TH) is an enzyme localized inside sympathetic neurotransmitter storage vesicles [58] that is used as a peripheral sympathetic marker [43,58]. The higher TH immunoreactivity observed (induced by undernutrition during foetal life) indicates the occurrence of a sympathetic hyperinnervation in MUN mesenteric arteries and is in accordance with a previous report in FPH induced by prenatal hypoxia, in which a sympathetic hyperinnervation in tibial arteries was described [59]. A denser innervation and higher NA content was also observed in SHR mesenteric arteries, in accordance with previous studies [54,60]. The larger innervation was associated with higher noradrenaline (NA) content and release, assessed by functional studies using [^3^H]-NA, which can be taken up into vesicles in sympathetic nerve terminals by a specific NA transporter [61,62]. The total tissue tritium content can be considered an indicator of the sympathetic innervation density [54]. The larger release of tritium upon electrical stimulation can be a consequence of the hyperinnervation observed. However, it may also reflect changes in the presynaptic machinery and regulation of noradrenaline release. We have previous evidence that these mechanisms are altered in both SHR [43,53,54] and MUN rats [63]. Our data also showed that the total tissue content of the MUN mesenteric artery was higher compared to CONTROL, indicating that the MUN nervous terminals can incorporate more NA. Our functional data showed a higher sympathetic output in both SHR and MUN rats, compared to their respective controls. While the difference in innervation was similar for both hypertensive models, the tritium outflow and NA content was relatively higher in MUN rats, suggesting an increased availability of NA in the synaptic cleft of sympathetic MUN nerves. Similar data was found in SHR mesenteric [54,64] and tail [65] arteries. Our data agrees with studies in other models of FPH showing that SNS activation seems to be increased [36,58,66,67,68,69]: it was described an increased circulating levels of NA in animal models of FPH [70,71,72] and in LBW humans [73].

An increased sympathetic neurotransmission can contribute to increase vascular tone and to the observed remodelling, which, in turn, can be one of the mechanisms behind hypertension development in MUN. When compared to SHR, the increased NA release observed was analogous to MUN, although SHR presented a smaller basal outflow. This data is in line with previous studies in SHR, in which a sympathetic hyperactivity was also described in the cerebral artery [74], tail artery [49] and mesenteric bed [52]. Morphometric analysis indicated that foetal undernutrition induces inward hypertrophic remodelling in the mesenteric artery, with the contribution of both media and adventitia layers, and the presence of vascular fibrosis, in agreement with data from other studies, in SHR [75] and MUN [76,77]. The MUN mesenteric artery exhibits a reduced lumen area as well as an increased media and adventitia layers compared to CONTROL, indicative of inward hypertrophic vascular remodelling. A similar profile of vascular wall changes between SHR and WKY was also observed. This type of remodelling is a characteristic of hypertension and can contribute to increase total peripheral vascular resistance [78]. In mesenteric arteries from rats exposed to undernutrition in utero, the same type of remodelling was also reported [79].

The hypertrophy observed in the media layer of MUN mesenteric artery can be due to cellular hypertrophy caused by vascular smooth muscle cell (VSMC) proliferation or growth. Sympathetic hyperinnervation was shown to be related to media hypertrophy in jejunal arteries of SHR, since an increase in the number of nerve fibres occurred before the development of hypertension or an increase in the thickness of the arterial media [79]. Such finding suggests that increased sympathetic activity possibly plays a causal role in the development of hypertension, through vascular remodelling. Our study proves that the remodelling process is relatively similar in MUN and SHR (inward hypertrophic).

MUN had higher SBP and DBP than CONTROL, confirming previous results with the same foetal model [11,45,80,81,82]. SHR also exhibited higher values of SBP compared to WKY, as shown in many studies [54,83,84,85]. It was not possible to compare the blood pressure directly between SHR and MUN male rats, since they have a control strain with different background. However, the current study indicates that foetal undernutrition induces a milder form of hypertension when compared to a genetic model of essential hypertension, with lower levels of blood pressure (around 50 mmHg difference for SHR-WKY and 25 mm Hg for MUN-SD control). Additionally, MUN rats did not show an elevated HR, which was detected in the SHR model. The lack of HR alterations of MUN rats is in line with other studies using maternal low protein [35,86] or low micronutrients, such as zinc [87] or vitamin B12 [88] diets during pregnancy. In SHR, the tachycardia probably reflects an alteration in baroreceptor regulation, as previously reported [86], which may contribute to the higher blood pressure in this strain. It should be considered that the blood pressure data was obtained under ketamine/medetomidine anaesthesia, which exerts an influence on the sympathetic nervous system: ketamine has been shown to reduce both exocytosis and NA uptake [89]; medetomidine has been reported to reduce noradrenaline outflow within the central nervous system, dampening the central sympathetic tone [90]. Given our findings of an increased sympathetic activity and innervation in both SHR and MUN rat, it is likely that the blood pressure in both models of hypertension was underestimated. Accordingly, previous studies in adult SHR assessed by tail cuff showed high blood pressure levels over 220 mmHg of SBP in awake animals [91], relatively to those reported in the present study under anaesthesia (SBP = 186 mmHg). In MUN rats, recent reports in awake 6-month-old rats showed SBP values of 163.1 mmHg [92], which are higher than those reported in the present study under anaesthesia (SBP = 151.1 mmHg).

In SHR rats, sex differences have been associated, among other causes, with increased sympathetic outflow due to dysfunctional regulation of presynaptic α-adrenoceptors in males [93]. Regarding the MUN model, we have reported that females counteract better the effects of foetal stress and do not develop hypertension in adult life [46]. It is important to know that these studies were performed under medetomidine/ketamine anaesthesia. However, recent reports have revealed that, in non-anesthetized rats, there is a tendency toward hypertension in 6-month-old MUN female rats, which is established and evident in old age [91]. Such data suggested that MUN females may also have increased blood pressure levels, and this could also explain the similarities in vascular remodelling between males and females [81]. Thus, the analysis of sex differences in MUN models deserves further attention.

## 5. Conclusions

**Figure 7:**
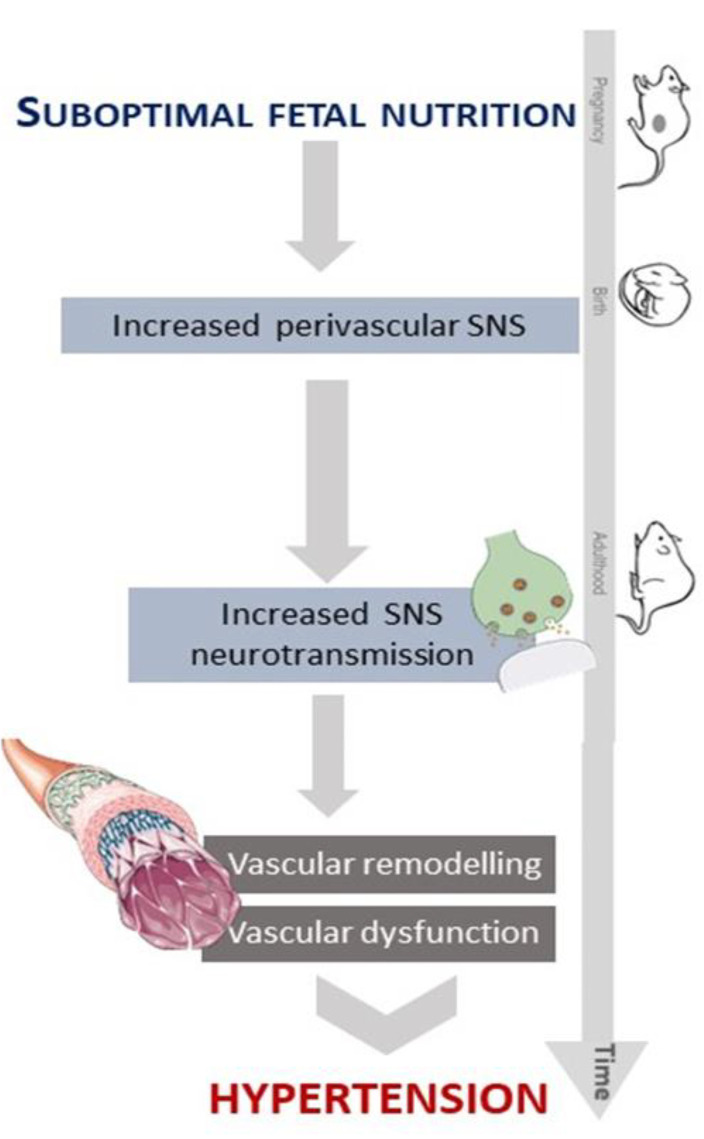
Effect of suboptimal foetal undernutrition on SNS and vascular wall from a rat FPH model.

In conclusion, and as depicted in Figure 7, elevated sympathetic neurotransmission in MUN and SHR supports that SNS plays an important role in the development of hypertension in both foetal programming and essential hypertension, which is in line with the neurogenic hypothesis of hypertension [94]. The similarities regarding sympathetic neurotransmission and remodelling in both models suggest that the increased sympathetic activity observed in FPH plays a causal role in the development of hypertension through vascular remodelling.

## Figures and Tables

**Table 1:** Blood Pressure and Heart Rate from CONTROL/MUN and WKY/SHR rats.

Mesenteric Artery	DBP (mmHg)	SBP (mmHg)	HR (Beat/min)	*n*
CONTROL	68.7 ± 4.2 ^†^	125.6 ± 5.1	258 ± 8	6
MUN	90.2 ± 4.5 *^#^	151.1 ± 4.1 *^#^	253 ± 9 ^#^	6
WKY	98.5 ± 5.5	133.1 ± 4.3	250 ± 10	6
SHR	140.3 ± 5.9 *	186.2 ± 8.3 *	318 ± 8 *	6

Four animal groups in the study: MUN, offspring from rats exposed to maternal undernutrition during pregnancy; CONTROL, offspring from mothers fed ad libitum during pregnancy; SHR, spontaneously hypertensive rats; WKY, Wistar Kyoto rats. SBP, systolic blood pressure; DBP, diastolic blood pressure; HR, heart rate. Values are mean ± s.e.m, from *n* rats from each group in study. Significant differences from the appropriate animal control: * *p* < 0.05; from the WKY model: ^†^ *p* < 0.05; from the SHR model: ^#^ *p* < 0.05.

**Table 2:** Vascular wall morphology changes associated with hypertension.

Mesenteric Artery	Ratio Media/Lumen	Ratio Adventitia/Lumen	*n*
CONTROL	0.626 ± 0.016	0.419 ± 0.057	6
MUN	0.989 ± 0.314 *	0.829 ± 0.303 *	6
WKY	0.307 ± 0.092	0.266 ± 0.112	6
SHR	0.519 ± 0.165 *	0.398 ± 0.187 *	6

Images were obtained from haematoxylin/eosin-stained arteries. Four animal groups in the study: MUN, offspring from rats exposed to maternal undernutrition during pregnancy; CONTROL, offspring from mothers fed ad libitum during pregnancy; SHR, spontaneously hypertensive rats; WKY, Wistar Kyoto rats. Values are mean ± s.e.m, from 6 rats from each group in study. Significant differences from the appropriate animal control: * *p* < 0.05.

**Table 3:** Basal tritium outflow (b_1_), electrically evoked tritium overflow (S_1_) and S_2_/S_1_ ratios from mesenteric arteries from CONTROL/MUN and WKY/SHR rats.

Mesenteric Artery	Basal Outflow (b_1_) (Fractional Rate Outflow; min^−1^)	Evoked Overflow (S_1_) (% of Tissue Tritium Content)	*n*
CONTROL	0.097 ± 0.008	0.220 ± 0.040	6
MUN	0.093 ± 0.006	0.347 ± 0.030 *	6
WKY	0.088 ± 0.006	0.265 ± 0.021	6
SHR	0.073 ± 0.006	0.381 ± 0.032 *	6

Tissues were stimulated twice at 30-min intervals (S_1_–S_2_; 100 pulses, 5 Hz, 1 ms, 50 mA): b_1_ refers to the 5-min period immediately before S_1_. The electrically evoked tritium overflow is expressed as a percentage of the tissue tritium content at the onset of stimulation. Basal tritium outflow (b_1_), electrically evoked tritium overflow (S_1_) from the four animal groups in study. MUN, offspring exposed to maternal undernutrition during pregnancy; CONTROL, offspring from mothers fed ad libitum during pregnancy; SHR, spontaneously hypertensive rats; WKY, Wistar Kyoto rats. Values are mean ± s.e.m, from 6 rats from each group in study. Significant differences from the appropriate animal control: * *p* < 0.05.

## Data Availability

The data presented in this study are available on request from the author (M.S.V.-R.).
